# Draft Genome Sequence of Calcium-Dependent *Paenibacillus* sp. Strain TCA20, Isolated from a Hot Spring Containing a High Concentration of Calcium Ions

**DOI:** 10.1128/genomeA.00866-14

**Published:** 2014-09-04

**Authors:** Shun Fujinami, Kiyoko Takeda-Yano, Takefumi Onodera, Katsuya Satoh, Motohiko Sano, Yuka Takahashi, Issay Narumi, Masahiro Ito

**Affiliations:** a Bio-Nano Electronics Research Centre, Toyo University, Kawagoe, Saitama, Japan; b Faculty of Agriculture, Tokyo University of Agriculture and Technology, Fuchu, Tokyo, Japan; c Cooperative Research Centre of Life Sciences, Kobe Gakuin University, Kobe, Hyogo, Japan; d Ion Beam Mutagenesis Research Group, Medical and Biotechnological Application Unit, Quantum Beam Science Center, Japan Atomic Energy Agency, Takasaki, Gunma, Japan; e Graduate School of Life Sciences, Toyo University, Itakura-machi, Gunma, Japan; f Faculty of Life Sciences, Toyo University, Itakura-machi, Gunma, Japan

## Abstract

Calcium-dependent *Paenibacillus* sp. strain TCA20 was isolated from a water sample of a hot spring containing a high concentration of calcium ions. Here, we report the draft genome sequence of this bacterium, which may be the basis for the research of calcium ion homeostasis.

## GENOME ANNOUNCEMENT

Calcium ions have an important role in transmitting signals not only in eukaryotes but also in bacteria (1). For example, the calcium ion concentrations of *Bacillus subtilis* cells are increased in the sporulation process (2). In addition, calcium efflux is essential for pathogenic bacteria (3). Intracellular calcium concentration is regulated by calcium influx and efflux systems (3–6). However, little is known about the calcium transporter of bacteria. It was expected that genomic analysis of a calcium ion-dependent bacterium would provide novel information about calcium ion transporters and calcium ion homeostasis. Here, we report the draft genome sequence of *Paenibacillus* sp. strain TCA20, which showed calcium-dependent growth. This bacterium was newly isolated from a water sample of Tsurumaki-Onsen, which is well known as a Japanese hot spring containing a high concentration of calcium ions (1,740 mg/liter), and this bacterium appeared to be most closely related to *Paenibacillus urinalis* based on partial 16S rRNA gene sequence identity.

The draft genome sequence of *Paenibacillus* sp. strain TCA20 totals 5,631,463 bp in length and is composed of 33 large contigs (>500 bp), obtained using the Roche GS Junior and assembled using the GS *de novo* Assembler version 2.7. Automatic annotation was performed using the Microbial Genome Annotation Pipeline (7), which predicted a total of 5,226 protein-coding genes. The product names of the predicted protein-coding genes were manually revised. tRNA detection was performed using the ARAGORN software (8), which predicted a total of 86 tRNAs.

In *B. subtilis*, a calcium-specific calcium/proton antiporter, ChaA (YfkE), and a P-type calcium-transporting ATPase, YloB, were identified as calcium transporters (5, 6). It was suggested that ChaA and YloB are important for calcium signaling in the sporulation or germination process in *Bacillus* species. The annotation of the draft genome sequence shows that *Paenibacillus* sp. strain TCA20 has a *chaA* gene and a gene that encodes a putative P-type calcium-transporting ATPase. In *Streptococcus pneumoniae*, the P-type calcium-transporting ATPase CaxP is used to avoid an accumulation of calcium ions in the eukaryotic host (3). In addition to the calcium signaling in the sporulation or germination process, these transporters may also be important for the growth of *Paenibacillus* sp. strain TCA20 under the high concentration of calcium ions.

The annotation of the draft genome sequence also shows that *Paenibacillus* sp. strain TCA20 has a set of *mrp* genes that encode multisubunit cation/proton antiporter-3 family proteins. It was reported that the Mrp antiporter of *Thermomicrobium roseum*, isolated from a hot spring, acts as a calcium/proton antiporter (9). The Mrp antiporter of *Paenibacillus* sp. strain TCA20 may also support calcium efflux.

However, these three transporters are also found in many calcium-independent bacteria. Further analysis is required to identify the calcium-dependent mechanism of *Paenibacillus* sp. strain TCA20.

### Nucleotide sequence accession numbers.

The draft genome sequence of *Paenibacillus* sp. strain TCA20 was deposited at DDBJ/EMBL/Genbank under the accession no. BBIW00000000. The version described in this paper is the first version, BBIW01000000.
